# Multidomain Lifestyle Intervention Strategies for the Delay of Cognitive Impairment in Healthy Aging

**DOI:** 10.3390/nu10101560

**Published:** 2018-10-21

**Authors:** Josef Toman, Blanka Klímová, Martin Vališ

**Affiliations:** 1 Department of Applied Linguistics, University of Hradec Kralove, Rokitanskeho 62, 500 03 Hradec Kralove, Czech Republic; josef.toman@uhk.cz; 2 Department of Neurology, University Hospital Hradec Kralove, Sokolska 581, 500 05 Hradec Kralove, Czech Republic; martin.valis@fnhk.cz

**Keywords:** multidomain intervention, lifestyle strategies, elderly people, cognitive impairment, randomized clinical trials

## Abstract

Present demographic changes demonstrate that the number of elderly people is growing at a frenetic pace. This shift in population consequently results in many social and economic problems, which burden the social and economic systems of countries. The aging process is associated with age-related diseases, the most common of which are dementia and Alzheimer’s disease, whose main symptom is a decline in cognitive function, especially memory loss. Unfortunately, it cannot be cured. Therefore, alternative approaches, which are cost-effective, safe, and easy to implement, are being sought in order to delay and prevent cognitive impairment. The purpose of this review was to explore the effect of multidomain lifestyle intervention strategies on the delay and/or prevention of cognitive impairment in healthy older individuals. The methods are based on a literature review of available sources found on the research topic in three acknowledged databases: Web of Science, Scopus, and PubMed. The results of the identified original studies reveal that multidomain lifestyle interventions generate significant effects. In addition, these interventions seem feasible, cost-effective, and engaging. Thus, there is a call for the implementation of effective lifestyle prevention programs, which would involve goal-setting and would focus on the prevention of crucial risk factors threatening the target group of elderly people, who are at risk of cognitive decline and dementia.

## 1. Introduction

Due to demographic changes, the number of the elderly people is growing at a frenetic pace [1]. For instance, in 2030, the number of elderly people aged 65+ years should reach 19%, which is 7% more than that in 2000 [2]. In developed countries, the number of older adults represents 24% of the population and it should rise to 33% by 2050 [3]. Thus, by 2050, the number of elderly people will outnumber the young population in most of these countries [4]. This shift in population consequently results in many social and economic problems [5], which burden the social and economic systems of countries. Therefore, governments attempt to develop appropriate measures and strategies which would delay the process of aging and prolong the active age of older individuals, as well as maintain their quality of life [6].

The aging process is associated with age-related diseases, the most common of which is dementia [7]. Dementia is a clinical syndrome, which is associated with cognitive decline, involving loss of memory and reasoning difficulties. It is also one of the main causes of incapability and dependency of older people [8,9]. Therefore, alternative approaches, which are non-invasive, cost-effective, safe, and easy to implement, are being sought in order to delay and prevent cognitive impairment. Research studies [10,11,12,13,14,15] indicate that there are several modifiable risk factors (i.e., low education, sedentary lifestyle, midlife obesity, midlife smoking, hypertension, diabetes, and midlife depression) which can be addressed to delay the onset of cognitive impairment through lifestyle interventions. They also report that multidomain lifestyle interventions in particular (e.g., References [15,16,17,18]) are more effective than just single ones (e.g., References [19,20,21]). In this study, multidomain lifestyle strategies are defined as interventions that intervene in at least two different domains, of which one has to be diet/nutritional intervention (with respect to the focus of the Special Issue) and the other intervention could include cognitive training intervention, physical exercise intervention, social activity intervention, and vascular risk management intervention.

The purpose of this review study was to explore the effect of multidomain lifestyle intervention strategies on the delay and/or prevention of cognitive impairment in healthy older individuals.

## 2. Methods

The methods are based on a literature review of available sources found on the research topic in three acknowledged databases: Web of Science, Scopus, and PubMed. The search was not limited by any time period, because multidomain lifestyle intervention studies started appearing around 2010. The literature search was conducted between 10 July 2018 and 10 September 2018 to identify published peer-reviewed articles in English. The collocated keywords were as follows: “multidomain intervention” AND “cognitive decline” AND “older people”, “multidomain intervention” AND “cognitive impairment” AND “older people”, “multidomain intervention” AND “cognitive impairment” AND “elderly”, “lifestyle intervention” AND “cognitive decline” AND “elderly”, “lifestyle intervention” AND “dementia” AND “older people”, “multidomain intervention” AND “dementia” AND “older people”, “multidomain intervention” AND “dementia” AND “elderly”, and “lifestyle intervention” AND “Alzheimer’s disease” AND “elderly”. The keywords were combined and integrated in database and journal searches. The terms used were searched using AND to combine the keywords listed and OR to remove search duplication where possible. References of retrieved articles were assessed for relevant articles that the authors’ searches may have missed.

From the database/journal searches, 489 titles/abstracts were retrieved. The majority of the studies were detected in the PubMed database (396 studies), followed by Web of Science (67 studies) and Scopus (26 studies). The titles and abstracts of some identified articles were then checked for relevance. Subsequently, the search was performed again, focusing on the occurrence of at least one keyword in the title or abstract, thereby significantly narrowing the selection. It provided the authors with a relevant entry-level file base. Altogether, 68 studies were found. Furthermore, three articles were identified from other available sources (i.e., web pages, conference proceedings, and books outside the scope of the databases described above). After removing duplicates and titles/abstracts unrelated to the research topic, 35 English-written studies remained. Of these, only 23 articles were relevant to the research topic. These studies were investigated in full and they were considered against the inclusion and exclusion criteria below. The inclusion criteria were as follows:

- The period of the publishing of the article was not limited;
- Only reviewed full-text studies in scientific journals in English were included;
- Only randomized controlled trials, cohort studies, or experimental/cross-sectional studies were involved and they had to include at least two interventions in different domains, of which one had to be diet/nutritional intervention;
- The primary outcome was aimed at the improvement in cognitive function;
- The subjects were healthy older individuals aged 50+ years.
- The exclusion criteria were as follows:
- The study was in a language other than English;
- The study did not involve healthy older individuals;
- The study protocols such as References [13,14,15], multidomain lifestyle interventions focusing on just one strategy [22], single lifestyle intervention studies [19,23,24], multidomain lifestyle studies that did not include diet intervention [25], multidomain lifestyle studies that did not focus on the improvement in cognitive function [26], and review studies (e.g., References [10,11,27,28]) were also excluded.

**Figure 1:**
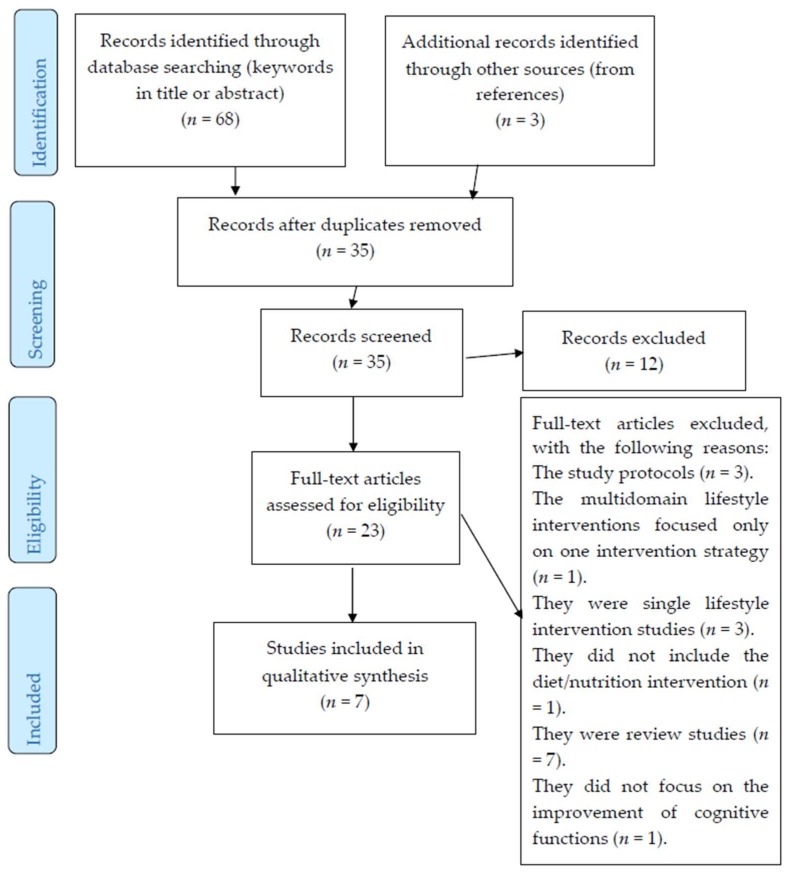
An overview of the selection procedure.

Based on these criteria, seven studies were involved in the final analysis. Figure 1 demonstrates the selection procedure.

## 3. Results

Altogether, seven original studies were eventually identified. Five were randomized controlled trials (RCTs) [16,17,18,29,30], one was a prospective cohort study [31], and one was a cross-sectional study [12]. Six originated in Europe (Finland, France, Sweden, and UK) [12,16,17,18,29,30] and one originated in USA [31]. The publishing date of the first study was 2009 [31], followed by the years of 2015 [16,30], 2017 [12,18], and 2018 [17,29]. Apart from one study [31] which involved only two intervention strategies (i.e., diet and physical exercises), all other studies included the three same intervention strategies: diet, cognitive training, and physical exercise. Moreover, some of them also comprised vascular risk management [16,17,18] or social activities [30], or even alcohol consumption [12]. References [16,17] were duplications of the same project, but with different aims.

The intervention period in the studies ranged from one year to 14 years. Overall, the sample sizes were quite big, ranging from 775 to 1680 healthy older individuals. Only one study [30] included 75 healthy subjects. The efficacy of the multidomain lifestyle interventions on the delay and/or prevention of cognitive impairment was assessed with standard neurological and neuropsychological tests, statistical analysis, and, in some cases, questionnaires [30,31] or observations [16]. The main strengths of the selected studies were the large sample sizes, duration of interventions, and standardized outcome measures. On the contrary, the limitations comprised the lack of patients in a clinical setting, as well as homogeneous healthy older population groups, a lack of statistical power, a lack of follow-up periods in most cases, and differences in methodologies, especially in the case of no RCT.

Apart from one study [29], findings of the detected studies showed that multidomain interventions were beneficial for the delay of cognitive impairment. They improved cognitive functioning, especially executive functioning, of older people, particularly those at high risk of dementia. Table 1 provides an overview of the main findings from the selected studies. They are summarized in alphabetical order based on first author.

## 4. Discussion

Apart from one study [29], the results from Table 1 reveal that the multidomain lifestyle interventions generated significant effects. This was confirmed by other research studies [10,32,33]. In addition, these interventions seem feasible, cost-effective, and engaging [30]. Thanks to a number of different activities, i.e., healthy diet, physical exercises, cognitive training, social activities, or vascular risk monitoring, they target several risk factors such as obesity, sedentary lifestyle, hypertension, or depression [10,16]. As Pope et al. [34] claim, adherence to a healthy lifestyle may directly protect against cognitive decline or may prevent diseases connected with cognitive impairment, such as vascular diseases. Therefore, the development of multidomain lifestyle intervention strategies, which contribute to the prevention of cognitive decline in healthy older individuals, as this review showed, is of cardinal importance [35]. This opinion is currently supported by the European Dementia Prevention Initiative, an investigator-initiated initiative of several groups involved in ongoing dementia prevention trials in Europe [36]. In fact, since 2017, this initiative spread to other continents and the reduplication of European projects (e.g., Finnish Geriatric Intervention Study to Prevent Cognitive Impairment and Disability (FINGER) study) will be performed in Asia, USA, or Australia [37].

Thus, there is a call for the implementation of effective lifestyle prevention programs, which would be goal-setting and would focus on the prevention of crucial risk factors threatening the target group of elderly people who are at risk of cognitive decline and dementia [38,39]. A recent study by Barbera et al. [40] on an internet-based counseling multidomain lifestyle intervention program for the prevention of cardiovascular disease and cognitive impairment in older adults, the so-called Healthy Aging through Internet Counseling in the Elderly (HATICE) trial, demonstrated that this is feasible and applicable internationally. However, these multidomain lifestyle intervention strategies should be integrated at an optimal level into the daily regime of healthy older individuals [34].

The limitations of this review consist of a small number of the selected studies on the research topic, single-type population and nationality groups, and, in particular, a lack of follow-up assessments. All these insufficiencies might generate overestimated conclusions of this review study [41,42]. Therefore, more RCTs are needed to prove the efficacy of multidomain lifestyle interventions on the delay and/or prevention of cognitive impairment.

## 5. Conclusions

Overall, on the basis of the results of this review study, the authors conclude that multidomain lifestyle interventions are effective in the delay and/or prevention of cognitive impairment in healthy older individuals. Nevertheless, people should start performing them regularly as early as midlife, so that they could have an impact on cognitive function in later life [28]. The results confirm that diet/nutrition, cognitive training, and physical exercise interventions are particularly effective in this sense. 

## Figures and Tables

**Table 1:** Overview of the seven selected studies on the efficacy of multidomain lifestyle intervention on the delay of cognitive impairment in healthy older individuals.

Author	Types of Multidomain Lifestyle Interventions and Their Frequency	Intervention Period	Number of Subjects	Main Findings	Limitations
Andrieu et al. [29] RCT(France)	Omega-3 supplementation (total daily dose of 800 mg docosahexanoic acid and up to 225 mg eicosapentanoic acid); multidomain intervention (nutritional and exercise counselling and cognitive training); omega-3 plus multidomain intervention or placebo with usual care; (2 h twice a week during the first 4 weeks and once a week for the following 4 weeks, and then once a month)	3 years	1680 elderly people with subjective memory complaints; age: 70+ years	Multidomain intervention and polyunsaturated fatty acids, either alone or in combination, had no significant effects on cognitive decline over 3 years in elderly people with memory complaints.	No blinded participants; low intensity of intervention; decreasing adherence with time
Clare et al. [12] Cross-sectional study(UK)	Cognitive activity; social engagement; physical activity; diet; alcohol consumption; smoking	2 years + 2 years of follow-up	2315 cognitively healthy participants; age: 65+ years	The findings indicated that participation in cognitive, social, and physical activity along with a healthy diet and light-to-moderate alcohol consumption may help maintain cognitive health in later life, together accounting for 20% (95% confidence intervals: 17–23%) of variance in cognitive test scores.	Causal relations could not be inferred; conceptually difficult to assess cognitive lifestyle and reserve
Clare et al. [30] RCT(UK)	Three groups: control (IC)—an interview in which information about activities and health was discussed; goal-setting (GS)—an interview in which they set behavior change goals relating to physical, cognitive, and social activity, health and nutrition; and goal-setting with mentoring (GM)—the goal-setting interview followed by bi-monthly telephone mentoring. The one-to-one interviews lasted for 90 min.	12 months	75 healthy elderly (IC—27 subjects; GS—24 subjects; GM—24 subjects); age: 50+ years	The results showed that, at 12-month follow-up, the two goal-setting groups increased their level of physical (effect size 0.37) and cognitive (effect size 0.15) activity relative to controls.	Men did not participate.
Ngandu et al. [16] RCT(Finland, Sweden)	Multidomain intervention (diet, exercise, cognition, and vascular risk management) and regular health advice; diet meetings and group discussions several times per year; exercises at least twice a week for 45 min; cognitive training 3 times a week for 10–15 min; vascular risk management meeting every three months.	2 years	1260 subjects: 631 subjects in the intervention group and 629 in the control group; mean age: 69.3 years	The findings indicated that a multidomain intervention could improve or maintain cognitive function in at-risk elderly people from the general population.	No patients in clinical settings
Rosenberg et al. [17] RCT(Finland, Sweden)	Multidomain intervention (diet, exercise, cognition, and vascular risk management) and regular health advice; diet meetings and group discussions several times per year; exercises at least twice a week for 45 min; cognitive training 3 times a week for 10–15 min; vascular risk management meeting every three months.	2 years	1260 subjects: 631 subjects in the intervention group and 629 in the control group; mean age: 69.3 years	The results showed that socio-demographics, socioeconomic status, cognition, cardiovascular factors, and cardiovascular comorbidity did not modify response to intervention (*p*-values for interaction >0.05).	A lack of statistical power
Scarmeas et al. [31] Prospective cohort study(USA)	Light (e.g., walking, golfing, or horse riding) to intense physical activities (e.g., aerobics, jogging, or playing handball); Mediterranean-type diet.	14 years	Two cohorts of 1880 elderly individuals with complete dietary and physical activity information; mean age: 76 years	Doing more physical activities per day and week and keeping the Mediterranean diet had benefits for the delay of cognitive decline. The results also revealed that the highest tertiles for both physical activity and Mediterranean-type diet were connected with a 61% to 67% lower risk of Alzheimer’s disease.	Reporting of physical activity was not measured; follow-up period was short; patients with mild cognitive impairment were excluded.
Sindi et al. [18] RCT(Finland)	Participants were randomly assigned to the lifestyle intervention (diet, exercise, cognitive training, and vascular risk management) and control (general health advice) groups.	2 years	775 healthy subjects (392 control, 383 intervention): age: 60–77 years	The findings of the intervention revealed that cognitive benefits were more pronounced with shorter baseline of leukocyte telomere length (LTL), particularly for executive functioning, indicating that the multidomain lifestyle intervention was especially beneficial among higher-risk individuals.	No detection of intervention effect by baseline LTL

## References

[1] Klimova B., Simonova I., Poulova P., Truhlarova Z., Kuca K. (2016). Older people and their attitude to the use of information and communication technologies—A review study with special focus on the Czech Republic (Older people and their attitude to ICT). Educ. Gerontol..

[2] Vafa K. Census Bureau Releases Demographic Estimates and Projections for Countries of the World. http://blogs.census.gov/2012/06/27/census-bureau-releases-demographic-estimates-and-projections-for-countries-of-the-world/.

[3] United Nations, Department of Economic and Social Affairs, Population Division (2013). World Population Ageing 2013.

[4] Statista Proportion of Selected Age Groups of World Population in 2017, by Region. https://www.statista.com/statistics/265759/world-population-by-age-and-region/.

[5] Klimova B., Maresova P., Valis M., Hort J., Kuca K. (2015). Alzheimer´s disease and language impairments: Social intervention and medical treatment. Clin. Interv. Aging.

[6] Marešová P., Klímová B., Kuča K. (2015). Alzheimer´s disease: Cost cuts call for novel drugs development and national strategy. Ceska. Slov. Farm..

[7] Klimova B. (2016). Use of the Internet as a prevention tool against cognitive decline in normal aging. Clin. Interv. Aging.

[8] Klimova B., Maresova P., Kuca K. (2016). Non-pharmacological approaches to the prevention and treatment of Alzheimer´s disease with respect to the rising treatment costs. Curr. Alzheimer Res..

[9] Klimova B., Valis M., Kuca K. (2017). Potential of mobile technologies and applications in the detection of mild cognitive impairment among older generation groups. Soc. Work Health Care.

[10] Mangialasche F., Kivipelto M., Solomon A., Fratiglioni L. (2012). Dementia prevention: Current epidemiological evidence and future perspective. Alzheimer’s Res. Ther..

[11] Michel J.P. (2016). Is it possible to delay or prevent age-related cognitive decline?. Korean J. Fam. Med..

[12] Clare L., Wu Y.T., Teale J.C., MacLeod C., Matthews F., Brayne C., Woods B., CFAS-Wales Study Team (2017). Potentially modifiable lifestyle factors, cognitive reserve, and cognitive function in later life: A cross-sectional study. PLoS Med..

[13] Hardman R.J., Kennedy G., Macpherson H., Scholey A.B., Pipingas A. (2015). A randomised controlled trial investigating the effects of Mediterranean diet and aerobic exercise on cognition in cognitively healthy older people living independently within aged care facilities: The Lifestyle Intervention in Independent Living Aged Care (LIILAC) study protocol [ACTRN12614001133628]. Nutr. J..

[14] Tussing-Humphreys L., Lamar M., Blumenthal J.A., Babyak M., Fantuzzi G., Blumenstein L., Schiffer L., Fitzgibbon M.L. (2017). Building research in diet and cognition: The BRIDGE randomized controlled trial. Contemp. Clin. Trial.

[15] Kivipelto M., Solomon A., Ahtiluoto S., Ngandu T., Lehtisalo J., Antikainen R., Bäckman L., Hänninen T., Jula A., Laatikainen T. (2013). The Finnish Geriatric Intervention Study to Prevent Cognitive Impairment and Disability (FINGER): Study design and progress. Alzheimers Dement..

[16] Ngandu T., Lehtisalo J., Solomon A., Levalahti E., Ahtiluoto S., Antikainen R., Bäckman L., Hänninen T., Jula A., Laatikainen T. (2015). A 2 year multidomain intervention of diet, exercise, cognitive training, and vascular risk monitoring versus control to prevent cognitive decline in at-risk elderly people (FINGER): A randomised controlled trial. Lancet.

[17] Rosenberg A., Ngandu T., Rusanen M., Antikainen R., Bäckman L., Havulinna S., Hänninen T., Laatikainen T., Lehtisalo J., Levälahti E. (2018). Multidomain lifestyle intervention benefits a large elderly population at risk for cognitive decline and dementia regardless of baseline characteristics: The FINGER trial. Alzheimers Dement..

[18] Sindi S., Ngandu T., Hovatta I., Kåreholt I., Antikainen R., Hänninen T., Levälahti E., Laatikainen T., Lindström J., Paajanen T. (2017). Baseline telomere length and effects of a multidomain lifestyle intervention on cognition: The FINGER randomized controlled trial. J. Alzheimers Dis..

[19] Danthiir V., Hosking D.E., Nettelbeck T., Vincent A.D., Wilson C., O’Callaghan N., Calvaresi E., Clifton P., Wittert G.A. (2018). An 18-mo randomized, double-blind, placebo-controlled trial of DHA-rich fish oil to prevent age-related cognitive decline in cognitively normal older adults. Am. J. Clin. Nutr..

[20] Sorman D.E., Sundstrom A., Ronnlund M., Adolfsson R., Nilsson L.G. (2014). Leisure activity in old age and risk of dementia: a 15-year prospective study. J. Gerontol. B. Psychol. Sci. Soc. Sci..

[21] Redick T.S., Shipstead Z., Harrison T.L., Hicks K.L., Fried D.E., Hambrick D.Z., Kane M.J., Engle R.W. (2013). No evidence of intelligence improvement after working memory training: A randomized, placebo-controlled study. J. Exp. Psychol. Gen..

[22] Lehtisalo J., Ngandu T., Valve P., Antikainen R., Laatikainen T., Strandberg T., Soininen H., Tuomilehto J., Kivipelto M., Lindström J. (2017). Nutrient intake and dietary changes during a 2-year multi-domain lifestyle intervention among older adults: Secondary analysis of the Finnish Geriatric Intervention Study to Prevent Cognitive Impairment and Disability (FINGER) randomised controlled trial. Br. J. Nutr..

[23] Külzow N., Witte A.V., Kerti L., Grittner U., Schuchardt J.P., Hahn A., Flöel A. (2016). Impact of omega-3 fatty acid supplementation on memory functions in healthy older adults. J. Alzheimers Dis..

[24] Brickman A.M., Khan U.A., Provenzano F.A., Yeung L.K., Suzuki W., Schroeter H., Wall M., Sloan R.P., Small S.A. (2014). Enhancing dentate gyrus function with dietary flavanols improves cognition in older adults. Nat. Neurosci..

[25] Verghese J., Lipton R.B., Katz M.J., Hall C.B., Derby C.A., Kuslansky G., Ambrose A.F., Sliwinski M., Buschke H. (2003). Leisure activities and the risk of dementia in the elderly. N. Engl. J. Med..

[26] Barreto P.S., Rolland Y., Cesari M., Dupuy C., Andrieu S., Vellas B., MAPT Study Group (2018). Effects of multidomain lifestyle intervention, omega-3 supplementation or their combination on physical activity levels in older adults: Secondary analysis of the Multidomain Alzheimer Preventive Trial (MAPT) randomised controlled trial. Age Ageing.

[27] Arab L., Sabagh M.N. (2010). Are certain life style habits associated with lower Alzheimer disease risk?. J. Alzheimers Dis..

[28] Klimova B., Valis M., Kuca K. (2017). Cognitive decline in normal aging and its prevention: A review on non-pharmacological lifestyle strategies. Clin. Interv. Aging.

[29] Andrieu S., Guyonnet S., Coley N., Cantet C., Bonnefoy M., Bordes S., Bories L., Cufi M.N., Dantoine T., MAPT Study Group (2017). Effect of long-term omega 3 polyunsaturated fatty acid supplementation with or without multidomain intervention on cognitive function in elderly adults with memory complaints (MAPT): A randomised, placebo-controlled trial. Lancet Neurol..

[30] Clare L., Nelis S.M., Jones I.R., Hindle J.V., Thom J.M., Nixon J.A., Cooney J., Jones C.L., Edwards R.T., Whitaker C.J. (2015). The Agewell trial: A pilot randomised controlled trial of a behaviour change intervention to promote healthy ageing and reduce risk of dementia in later life. BMC Psychiatry.

[31] Scarmeas N., Luchsinger J.A., Schupf N., Brickman A.M., Cosentino S., Tang M.X., Stern Y. (2009). Physical activity, diet, and risk of Alzheimer disease. JAMA.

[32] Barnard N.D., Bush A.I., Ceccarelli A., Cooper J., de Jager C.A., Fraser G., Fraser G., Kesler S., Levin S.M., Lucey B. (2014). Dietary and lifestyle guidelines for the prevention of Alzheimer’s disease. Neurobiol. Aging.

[33] Barnes D.E., Yaffe K. (2011). The projected effect of risk factor reduction on Alzheimer’s disease prevalence. Lancet Neurol..

[34] Pope S.K., Shue V.M., Beck C. (2003). Will a healthy lifestyle help prevent Alzheimer’s disease?. Annu. Rev. Public Health.

[35] Polidori M.C., Nelles G., Pientka L. (2010). Prevention of dementia: Focus on lifestyle. Int. J. Alzheimers Dis..

[36] EDPI. http://www.edpi.org/.

[37] ALZFORUM. https://www.alzforum.org/news/conference-coverage/new-dementia-trials-test-lifestyle-interventions.

[38] Kulmala J., Ngandu T., Kivipelto M. (2018). Prevention matters: Time for global action and effective implementation. J. Alzheimer’s Dis..

[39] Winblad B., Amouyel P., Andrieu S., Ballard C., Brayine C., Brodaty H., Cedazo-Minguez A., Dubois B., Edvardsson D., Feldman H. (2016). Defeating Alzheimer’s disease and other dementias: A priority for European science and society. Lancet.

[40] Barbera M., Mangialasche F., Jongstra S., Guillemont J., Ngandu T., Beishuizen C., Coley N., Brayne C., Andrieu S., Richard E. (2018). Designing an Internet-Based Multidomain Intervention for the prevention of cardiovascular disease and cognitive impairment in older adults: The HATICE trial. J. Alzheimer’s Dis..

[41] Melby-Lervag M., Hulme C. (2016). There is no convincing evidence that working memory training is effective: A reply to Au et al. (2014) and Karbach and Verhaeghen (2014). Psychon. Bull. Rev..

[42] Melby-Lervag M., Hulme C. (2013). Is working memory training effective? A meta-analytic review. Dev. Psychol..

